# Temporomandibular Disorders in Professional Scuba Divers—A Cross-Sectional Study

**DOI:** 10.3390/dj13090434

**Published:** 2025-09-21

**Authors:** Ivica Pelivan, Joško Viskić, Nikša Dulčić

**Affiliations:** 1 Department of Removable Prosthodontics, School of Dental Medicine, University of Zagreb, Gundulićeva ulica 5, HR-10000 Zagreb, Croatia; pelivan@sfzg.unizg.hr; 2 Department of Fixed Prosthodontics, School of Dental Medicine, University of Zagreb, Gundulićeva ulica 5, HR-10000 Zagreb, Croatia; viskic@sfzg.unizg.hr

**Keywords:** temporomandibular disorders, TMD, scuba diving, RDC/TMD protocol

## Abstract

**Objectives:** Most of the published literature on this topic argues about painful teeth, masticatory muscles, and/or temporomandibular joints in scuba divers. This study aimed to determine the possible differences in the prevalence of TMD between the diver population and the general population. **Methods:** This was a cross-sectional epidemiological study. The standardized RDC/TMD protocol was used for both the study and the control group. A total of 84 individuals were randomly selected to participate in the study: 55 divers (study group) and 29 individuals from the general population (control group). **Results:** There was a significant difference in the group 2 and group 3 RDC/TMD Axis I diagnoses between the study and the control group. Logistic regression analysis showed that the diver population was 15.8 times more likely to develop a group 2 left joint and 12 times more likely to develop a group 3 right joint diagnoses than the general population. The RDC/TMD Axis II diagnoses were also significantly higher in the study group than in the control group, whereby the divers were considerably older and predominantly male. **Conclusions:** A higher prevalence of group 2 (disk displacements) and group 3 (other common joint disorders) diagnoses was found in the divers. However, these results should be taken considering the limitation that there was a lack of age and gender matching between the groups, which can cause confounding.

## 1. Introduction

Temporomandibular disorders (TMD) are a complex group of musculoskeletal conditions that affect the temporomandibular joint (TMJ), masticatory muscles, and associated structures [1]. Recent epidemiological studies have revealed significant variations in TMD prevalence globally, with estimates ranging from 5% to 12% in the general population, although some studies have reported prevalence rates as high as 31%, depending on the diagnostic criteria employed [2,3]. The multifactorial etiology of TMD encompasses biomechanical, psychosocial, genetic, and behavioral components, making it one of the most challenging orofacial pain conditions to effectively diagnose and manage [4].

Contemporary research has identified several etiological factors contributing to TMD development, including oral parafunctional behaviors, psychological distress, genetic predisposition, and occupational risk factors [5]. Oral overuse behaviors, such as bruxism, clenching, nail biting, and excessive gum chewing, have been strongly associated with painful TMD, with studies demonstrating significant correlations between these behaviors and myofascial pain and arthralgia [6]. Šimunović et al. revealed that 42% of participants exhibited at least one TMD symptom, with pencil or nail biting being the most common parafunction. These parafunctions and Class II malocclusion significantly increase the likelihood of TMD symptoms [7]. The psychological dimension of TMD has gained considerable attention, with anxiety, depression, and somatization frequently observed in patients with TMD, suggesting a complex interplay between psychological factors and pain perception [5].

The relationship between occupational factors and TMD development has received increasing attention in the recent literature, particularly focusing on activities requiring sustained jaw positioning or repetitive mandibular movements. Professional groups at elevated risk for TMD include musicians, especially wind instrument players, and occupations that require prolonged oral appliance use, like scuba divers [8,9].

Professional scuba divers represent a unique occupational group in various branches of the economy, such as in tourism, underwater construction, fishing, shipbuilding, and sea-based oil platforms [10,11]. Despite its attractiveness, underwater presents a potentially aggressive environment characterized by increasing hydrostatic pressure, poor visibility, low temperatures, and the necessity of maintaining prolonged jaw positions while using diving equipment [12]. This may contribute to the development of various medical disorders in divers, such as middle and inner ear barotrauma, decompression sickness, otologic disturbances including external otitis, exostosis of the ear canal, hearing loss, and vertigo [13,14]. Pressure changes may cause barodontalgia (pressure-induced toothache) and odontocrexis (loosening or cracking of restorations or teeth) [15].

Previous studies on TMD in scuba divers have predominantly relied on questionnaire-based assessments with little or no clinical evidence [16]. The Research Diagnostic Criteria for TMD (RDC/TMD) was developed by Dworkin and La Resche in 1992 as a protocol for clinical research and categorization of TMD [17]. This protocol was developed as a biaxial system. Axis I is a clinical physical examination protocol, and Axis II is a biobehavioral questionnaire designed to assess physiological distress and psychosocial dysfunction. A combination of the two axes provides a complete picture of the physical and mental state of an individual and possible underlying causes of TMD. Furthermore, this protocol is sensitive to disregarding disorders that are not clinically significant or have not yet been fully described and understood.

Recent advances in TMD diagnostic approaches have emphasized the importance of evidence-based assessment protocols and the integration of new diagnostic technologies, including advanced imaging techniques, biomarker analysis, and comprehensive pain assessment tools [18].

To our knowledge, limited research has specifically examined TMD prevalence of divers using standardized diagnostic protocols, such as the RDC/TMD. Previous studies on TMD in SCUBA divers have predominantly relied on questionnaire-based assessments with minimal clinical validation, highlighting the need for more rigorous epidemiological investigations in this specialized population.

The present cross-sectional epidemiological study employs the standardized RDC/TMD protocol to evaluate TMD prevalence and characteristics in professional divers compared with a general population control group [19]. The null hypothesis of this study assumes that there is no difference between the prevalence of temporomandibular disorders in divers and in the general population.

## 2. Materials and Methods

This study was approved by the Ethics Committee of the School of Dental Medicine, University of Zagreb, and it was conducted in the summer of 2021 and 2022.

A total of 84 participants were enrolled in the study, and they all provided informed consent in accordance with the Declaration of Helsinki. The participants were selected from a sampling frame defined by several inclusion and exclusion criteria by means of random number generator software. The refusal rate was low, and those who refused participation were replaced by the next randomly selected individual from the list to maintain sample representativeness.

The study group included 55 professional scuba divers (48 men and 7 women) aged 24 to 65 years (mean age 36.7 ± 1.2 years) from two diving centers, two underwater construction firms, and one sea-based tuna fish farm located on the Adriatic Sea in Croatia. The control group included 29 participants from the general population (16 men and 13 women), aged 25 to 40 years (mean age 30.1 ± 0.9 years).

Individuals with systemic, rheumatic, neurologic, endocrine, or autoimmune diseases, as well as recent jaw trauma or surgery, radiation treatment to the head and neck, non-TMD orofacial pain disorders, pregnancy, use of narcotic pain medication or certain drugs affecting muscle or pain assessment, language barriers, or mental incompetence preventing participation, were excluded from the study.

The RDC/TMD protocol was used as the standard for diagnosing temporomandibular disorders (TMD) in both the study group and the control group. Participants from both groups underwent a clinical physical examination according to the Axis I protocol. Depending on the signs and symptoms of TMD they showed, their diagnoses were classified into three groups: group 1 (muscle disorders), group 2 (disk displacement), and group 3 (other common joint disorders). Assessment according to the Axis II protocol was performed based on the completed biobehavioral questionnaire. Psychosocial factors contributing to or resulting from TMD were examined, and the diagnoses of depression level, nonspecific physical symptoms with pain, and nonspecific physical symptoms without pain were established.

The study was conducted by two researchers (J.V. and I.P.), who completed specialized education and training for this purpose. Cohen’s kappa test was applied to assess reproducibility. Altogether, twenty randomly selected participants from both the study group and the control group for each researcher were retested one week after the first examination. Randomization was performed using a computer-generated sequence with a kappa value threshold at κ > 0.75.

The normality of the distribution of continuous variables was checked using the Shapiro–Wilk test, and the homogeneity of variances was assessed using Levene’s test. As both the data distributions were asymmetric and could not be normalized by any transformation of data, and variances were not homogenous, the nonparametric Mann–Whitney U test was used to test the differences in continuous data between the study group and the control group. For descriptive statistics, the median and interquartile range were used because they provide a better description of asymmetric distributions than the mean and standard deviation. Chi-square and Fisher’s exact tests were used to analyze differences in frequencies between the study group and the control group.

A multiple logistic regression model was used to establish the correlation between TMD signs and symptoms in the study group and the control group. The presence of a particular TMD sign or symptom was used as a dichotomized variable (0 = absent, 1 = present). Age, depression level, and nonspecific physical symptoms with or without pain were included in the analyses as continuous independent variables. Chronic pain was used as the ordinal variable. The effect of gender was also considered an independent dichotomized variable (0 = female, 1 = male).

The backward stepwise method of binary logistic regression based on likelihood ratio statistics was used to select variables that fit the criteria for multiple regression models. The significance of the effects in the final model was determined using Wald statistics, and it was checked by means of a likelihood ratio test. Goodness-of-fit chi-square statistics and Nagelkerke’s pseudo R2 were used to assess how well the model fits the data. Odds ratios (OR) with 95% confidence interval limits (CIL) were used to measure the strength of the association between the presence of a factor and the occurrence of an event, indicating statistically significant relationships if both values were either greater or less than 1. This study was designed as an exploratory investigation to detect large effect sizes based on preliminary clinical observations. Post hoc power analysis was conducted using standard formulas for comparing two proportions, with statistical power calculated for α = 0.05 and the observed effect sizes. All analyses were performed using statistical software (SPSS, Release 30.0; SPSS Inc., Chicago, IL, USA). Statistical significance was set at *p* < 0.05.

## 3. Results

The study group of scuba divers had a significantly higher prevalence of group 2 and group 3 RDC/TMD Axis I diagnoses in the right and left TMJ than the control group. A significantly higher degree of chronic pain was found in the study group in relation to the control group. Odds ratios ranged from 6 to 14.8 (Table 1).

The divers had significantly higher values of Axis II diagnoses than the control group. However, they were older than the control group participants, and a statistically significant difference was found in their age (Table 2).

Furthermore, a more precise logistic regression analysis showed a significant difference between the study and control groups only in group 2 left joint and group 3 right joint diagnoses. The divers were older and predominantly male, and diving represented a greater risk for the development of these diagnoses (Table 3).

Post hoc power analysis revealed adequate statistical power (>80%) for the primarily significant outcomes: group 2 left joint (86.0% power), group 3 right joint (95.0% power), group 3 left joint (84.8% power), and chronic pain (88.9% power). However, the study was underpowered for group 1 muscle TMD (6.9% power), which may explain the non-significant findings in this category.

## 4. Discussion

The findings of this study demonstrated a significantly higher prevalence of specific TMD subtypes, particularly in group 2 (disk displacement) and group 3 (other common joint disorders) diagnoses according to the RDC/TMD protocol. These results align with the contemporary understanding of TMD etiology and the biomechanical demands associated with professional diving. Early findings of diving-related TMD revealed possible equipment-related musculoskeletal complications, which led to improvements in mouthpiece design and fitting [12]. However, our findings suggest that state-of-the-art diving equipment and practices continue to pose significant TMD risk, indicating a need for further technological and procedural innovations.

### 4.1. Scuba Diving and TMD

The current understanding of TMD etiology emphasizes the influence of sustained parafunctional activities and altered mandibular positioning in the development of TMD. The use of a diving mouthpiece causes prolonged mandibular protrusion and sustained masticatory muscle contraction and creates biomechanical conditions similar to those observed in other occupational TMD risk groups. Recent systematic reviews identified occupational factors as significant contributors to TMD development, with particular emphasis on occupations with activities requiring sustained, non-neutral jaw positioning, like in scuba divers [4].

Pain in the masticatory muscles and/or temporomandibular joints (TMJ) may result from occlusion imbalance or overexertion of the TMJ and muscles [20]. Mandibular protrusion and biting forces on the anterior occlusion during diving can cause pain and dysfunction, particularly in divers with bruxism [21]. This effect is due to a lack of posterior support and uneven loading within the TMJ and associated musculature [22]. While standard divers’ mouthpieces also increase the risk of TMD [22], several authors concluded that mouthpieces should be custom-made to distribute forces more evenly. However, custom-made mouthpieces are not commonly used, and TMD is frequently found in 24–68% of divers [18,23,24,25]. Ozturk et al. investigated 97 divers who experienced pain around the temporomandibular area, 14 of whom were diagnosed with TMD. Furthermore, TMD was significantly more common in inexperienced divers than in experienced divers, and the most prevalent symptom was increased effort required to grip the mouthpiece [22]. TMJ tenderness and trigger point activation were the most obvious physical signs of TMD. Thirteen divers showed improvement after treatment [22].

Lobbezzoo et al. [26] evaluated 536 scuba divers using a specific questionnaire and found that 485 were free of any TMD pain before they began diving. However, TMD pain was found in 214 participants (44.1%), which was significantly influenced by clenching, warm water, biting on the mouthpiece, and the perceived quality of the mouthpiece. The same authors also concluded that diving in cold water may protect against TMD pain [26]. Hobson studied 74 divers and reported that TMD unrelated to diving was increased by the use of a mouthpiece and that divers’ assessment of muscle tension and comfort while using a mouthpiece was a good predictor of TMD occurrence [20].

### 4.2. Axis I and Axis II Diagnoses

The main finding of this study was a higher prevalence of group 2 and 3 Axis 1 diagnoses in the study group than in the control group. Group 2 diagnoses comprise disk displacement with or without reduction, which can result from macro- or microtrauma. In the case of scuba diving-associated TMD, the main cause was microtrauma. The established 15.8-fold increased likelihood of developing group 2 diagnoses in the left joint and 12-fold increased risk of group 3 diagnoses in the right joint suggest a complex interaction between occupational factors and TMD pathogenesis. Recent research has established the multifactorial nature of disk displacement, identifying biomechanical overload, altered joint lubrication, and sustained non-physiological jaw position as its primary contributing factors [27]. The asymmetric distribution of TMD findings between the left and right TMJ observed in the present study may reflect differential loading patterns associated with mouthpiece positioning and individual diving habits. TMJ overload can lead to an uncontrolled production of reactive oxygen species that can cause oxidative stress [28,29] and consequent changes in condyle/disk mechanics and disk displacement [30,31]. Mechanical TMJ overload and disk displacement are the most common causes of TMJ arthritis development. Arthritis belongs to group 3 diagnoses, together with arthralgia and arthrosis [32]. A higher prevalence of these disorders in the study group in relation to the control group correlates with a higher prevalence of group 2 diagnoses.

Group 1 Axis 1 diagnosis of muscle TMD is a significant component of diving-related TMD, which is mainly treated with physiotherapy, occlusal appliance therapy, and behavioral modification techniques [33]. However, the unique occupational demands of professional divers may require specialized treatment modifications to address specific biomechanical challenges associated with mouthpiece use and underwater activity.

The values of Axis II diagnoses in the study group show increased psychological distress, which highlights the bidirectional relationship between psychological factors and TMD symptoms. Zlendić et al. also found strong associations between psychological distress, pain perception, and TMD severity [5]. These findings suggest that certain individuals may be predisposed to developing TMD when exposed to occupational risk factors, such as those encountered in professional diving.

### 4.3. Recent Research and Clinical Implications

Recent developments in TMD diagnostic technology, including advanced imaging techniques, dynamic MRI, and cone-beam computed tomography, provide detailed visualization of the temporomandibular joint structures and function and enable more precise diagnosis and individualized treatment according to specific patient characteristics [18]. New therapeutic methods, including advanced physiotherapy techniques, pharmacological interventions targeting specific pain pathways, and minimally invasive surgical procedures, have shown promising outcomes in treating various TMD subtypes, including myogenous pain [18]. For occupation-specific TMD cases, such as those found in the divers in this study, treatment strategies should address both the underlying pathophysiology and specific occupational risk factors that cause TMD.

The integration of contemporary TMD research with occupational health principles suggests that routine TMD screening should be incorporated into occupational health programs for professional divers. Early identification of TMD signs and symptoms, combined with evidence-based preventive interventions, may significantly reduce the risk of chronic TMD and its associated disabilities. Healthcare providers working with populations of divers should implement comprehensive assessment protocols and develop specialized management strategies that address the occupational risk factors associated with professional diving [34,35]. Additionally, technological advances in diving equipment design and promotion of individual mouthpiece systems may offer promising solutions for reducing occupational TMD risk while maintaining diving safety and performance standards.

### 4.4. Study Limitations and Further Research

The limitations of the current study include a relatively small number of participants, both in the study group and the control group, a lack of age and gender matching of participants in the control group, and a cross-sectional study design, which prevents the determination of causal relationships between diving activities and TMD development. We also acknowledge that the wide confidence intervals represent a limitation of our current study design and sample size. The sample of 84 participants limited the ability to detect small to moderate effects (OR < 4), and resulted in wide confidence intervals for some estimates. The study was adequately powered for detecting large effects found in disk displacement and TMJ arthralgia, but it was underpowered for smaller effects, particularly in connection with muscle disorders. Future studies should use larger sample sizes (200–300 participants per group) to detect moderate effects and provide more precise estimates.

Despite sample size limitations, the large effects detected (OR 6–15) represent clinically significant increases in TMD risk that require occupational health interventions. The 29–35% prevalence of disk displacement and joint arthralgia in divers compared to 3–7% in the control group represents a substantial occupational health concern requiring further investigation and preventive measures. Future longitudinal studies with larger cohorts are needed to establish temporal relationships and identify specific diving-related risk factors for TMD. Furthermore, as the age and gender differences between the groups in this study may have influenced the results, it is important for future studies to include age- and gender-matched participants in both the study group and the control group.

Although the standardized RDC/TMD protocol employed in this study provides a comprehensive framework for comparative research across different occupational populations, an updated Croatian version of the DC/TMD protocol could be the tool of choice for further research [36]. It was used in a recent study by Vrbanović et al., and it showed that participants with frequent oral parafunctions, regardless of pain level, had significantly higher values for somatosensory amplification, anxiety, depression, and hypervigilance [37].

Future research should focus on longitudinal studies to establish causal relationships between diving activities and TMD development. Besides investigating genetic TMD susceptibility in divers, additional factors, such as systemic diseases, specific genetic polymorphisms associated with pain sensitivity, inflammatory response, tissue repair mechanisms, and their interactions, should be explored, and innovative preventive strategies should be developed [38].

## 5. Conclusions

Axis II diagnoses according to the RDC/TMD protocol represent the psychological profile of the patient. Although the psychological profiles of participants with TMD could not be defined in the present study due to its limitations, increased anxiety, depression, and somatization were found. Therefore, it is possible to assume that the Axis II diagnoses in the study group of divers are related to the increased prevalence of group 2 and group 3 Axis I diagnoses. Within the limitations of this study, we conclude that there is a difference between the prevalence of temporomandibular disorders in scuba divers and in the general population, but there is a need for further investigation to gather more data through occupational screening in scuba divers.

## Figures and Tables

**Table 1:** Differences in the variables of the RDC/TMD between the study and control groups.

		Study		Control				
		*n*	(%)	*n*	(%)	*p* *	OR	95% CI
Group 1	negative	47	85.5%	26	89.7%			
	positive	8	14.5%	3	10.3%	0.741	1.5	0.4–6.1
Group 2 left	negative	39	70.9%	28	96.6%			
	positive	16	29.1%	1	3.4%	0.004	11.5	1.4–91.8
Group 2 right	negative	38	69.1%	27	93.1%			
	positive	17	30.9%	2	6.9%	0.014	6	1.3–28.3
Group 3 left	negative	36	65.5%	27	93.1%			
	positive	19	34.5%	2	6.9%	0.007	7.1	1.5–33.2
Group 3 right	negative	36	65.5%	28	96.6%			
	positive	19	34.5%	1	3.4%	0.001	14.8	1.9–117.2
Classification of chronic pain	0	32	58.2%	26	89.7%			
	1	21	38.2%	3	10.3%			
	2	2	3.6%			0.012		

* Fisher’s exact test.

**Table 2:** Differences in age and Axis II diagnoses between the study and control groups.

		*N*	Median	Interquartile Range	min	max	*p* *
Age	study	55	34	30–42	24	65	
	control	29	28	27–35.5	25	40	0.0009
Depression level	study	55	0.2	0.1–0.6	0	1	
	control	29	0.15	0.05–0.35	0	0.8	0.191
Nonspecific physical symptoms with pain	study	55	0.25	0.083–0.5	0	1.08	
	control	29	0.083	0.041–0.417	0	0.917	0.047
Nonspecific physical symptoms without pain	study	55	0.143	0–0.429	0	1.14	
	control	29	0	0–0.286	0	0.571	0.041

* Mann–Whitney U test.

**Table 3:** Differences between study and control group and RDC/TMD diagnoses including effects of age and gender (0 = female, 1 = male) assessed by logistic regression model.

Dependent Variable	Independent Variable	Logistic Coefficient	Standard Error	Wald Statistic	*p*	OR (95% CI)
Divers	Age	0.12	0.048	6.16	0.013	1.1 (1.0–1.2)
	Gender (male)	1.39	0.700	3.92	0.048	4.0 (1.0–15.8)
	Group 2 left	2.76	1.187	5.40	0.020	15.8 (1.5–161.9)
	Group 3 right	2.49	1.150	4.68	0.031	12.0 (1.3–114.7)

R^2^ = 0.35. (Cox and Snell Pseudo R Square)—only significant variables are shown.

## Data Availability

The data presented in this study are available upon request from the corresponding author.

## Abbreviations

The following abbreviations are used in this manuscript:

SCUBA	Self-Contained Underwater Breathing Apparatus
TMD	Temporomandibular Disorders
TMJ	Temporomandibular Joint
RDC/TMD	Research Diagnostic Criteria for Temporomandibular Disorders
DC/TMD	Diagnostic Criteria for Temporomandibular Disorders

## References

[1] Klasser G.D., Murchison D.F. (2025). Overview of Temporomandibular Disorders (TMDs). MSD Manual Professional Edition.

[2] Valesan L.F., Da-Cas C.D., Réus J.C., Hallak J.E.C., Boff B., de Oliveira M.S.R., Teixeira A.L., D’Orsi E. (2021). Prevalence of Temporomandibular Joint Disorders: A Systematic Review and Meta-Analysis. Clin. Oral Investig..

[3] Zieliński G., Pająk-Zielińska B., Ginszt M. (2024). A Meta-Analysis of the Global Prevalence of Temporomandibular Disorders. J. Clin. Med..

[4] Warzocha J., Gadomska-Krasny J., Mrowiec J. (2024). Etiologic Factors of Temporomandibular Disorders: A Systematic Review of Literature Containing Diagnostic Criteria for Temporomandibular Disorders (DC/TMD) and Research Diagnostic Criteria for Temporomandibular Disorders (RDC/TMD) from 2018 to 2022. Healthcare.

[5] Zlendić M., Vrbanović Đuričić E., Gall Trošelj K., Tomljanović M., Vuković Đerfi K., Alajbeg I., Alajbeg I.Z. (2025). Genetic, Psychological, and Behavioural Factors Associated with Subtypes of Pain-Related Temporomandibular Disorders. Biomedicines..

[6] Barbosa T.S., Miyakoda L.S., Pocztaruk R.L., Rocha C.P., Gavião M.B.D. (2021). Oral overuse behaviours are associated with painful temporomandibular disorders: A cross-sectional study. J. Oral Rehabil..

[7] Šimunović L., Lapter Varga M., Negovetić Vranić D., Čuković-Bagić I., Bergman L., Meštrović S. (2024). The Role of Malocclusion and Oral Parafunctions in Predicting Signs and Symptoms of Temporomandibular Disorders—A Cross-Sectional Study. Dent. J..

[8] Pihut M., Orczykowska M., Gala A. (2022). Risk Factors for the Development of Temporomandibular Disorders Related to the Work Environment—A Literature Review and Own Experience. Folia Med. Cracov..

[9] Sionek-Wręga I., Wręga J. (2024). Oral Health Complications of SCUBA Diving. Qual. Sport..

[10] Joiner J.T. (2001). NOAA Diving Manual: Diving for Science and Technology.

[11] Azizi M.H. (2011). Ear disorders in scuba divers. Int. J. Occup. Envirom. Med..

[12] Pinto O.F. (1966). Temporomandibular joint problems in underwater activities. J. Prosthet. Dent..

[13] Eichhorn L., Leyk D. (2015). Diving medicine in clinical practice. Dtsch. Arztebl. Int..

[14] Strutz J. (2008). Otorhinolaryngologische Erkrankungen beim Tauchen. HNO.

[15] Sheper W.A., Lobbezzoo F., Eijkman M.A. (2005). Oral problems in divers. Ned. Tijdschr. Tandheelkd..

[16] Branco C., Almeida A.M., Cebola P., Godinho C. (2021). Temporomandibular disorders in scuba divers: A systematic review. Ann. Med..

[17] Dworkin S.F., LeResche L. (1992). Research diagnostic criteria for temporomandibular disorders: Review, criteria, examinations and specifications, critique. J. Craniomandib. Disord..

[18] Moxley B., Stevens W., Sneed J., Pearl C. (2023). Novel diagnostic and therapeutic approaches to temporomandibular dysfunction: A narrative review. Life.

[19] Alolaiwi L.A., Alzahrani F.A., AlShammari S.A. (2025). Diving into discomfort: Orofacial pain dynamic—A systematic review. Front. Public Health.

[20] Hobson R.S. (1991). Temporomandibular dysfunction syndrome associated with scuba diving mouthpieces. Br. J. Sports Med..

[21] Grant S.M., Johnson F. (1998). Divers mouth syndrome: A report of two cases and construction of custom-made regulator handpiece. Dent. Update.

[22] Oztürk Ö., Tek M., Seven H. (2012). Temporomandibular disorders in scuba divers—An increased risk during diving certification training. J. Craniofac. Surg..

[23] Zadik Y., Drucker S. (2011). Diving dentistry: A review of the dental implications of scuba diving. Aust. Dent. J..

[24] Aldridge R.D., Fenlon M.R. (2004). Prevalence of temporomandibular dysfunction in a group of scuba divers. Br. J. Sports Med..

[25] Koob A., Ohlmann B., Gabbert O., Klingmann C., Rammelsberg P., Schmitter M. (2005). Temporomandibular disorders in association with scuba diving. Clin. J. Sport Med..

[26] Lobbezoo F., van Wijk A.J., Klingler M.C., Ruiz Vicente E., van Dijk C.J., Eijkman M.A. (2014). Predictors for the development of temporomandibular disorders in scuba divers. J. Oral Rehabil..

[27] Mercuri L.G. (2023). Temporomandibular Joint Facts and Foibles. J. Clin. Med..

[28] Nitzan D.W., Etsion I. (2002). Adhesive force: The underlying cause of the disc anchorage to the fossa and/or eminence in the temporomandibular joint—A new concept. Int. J. Oral Maxillofac. Surg..

[29] Nitzan D.W., Goldfarb A., Gati I., Kohen R. (2002). Changes in the reducing power of synovial fluid from temporomandibular joints with “anchored disc phenomenon”. J. Oral Maxillofac. Surg..

[30] Stegenga B., de Bont L.G., Boering G., van Willigen J.D. (1991). Tissue responses to degenerative changes in the temporomandibular joint: A review. J. Oral Maxillofac. Surg..

[31] Dijkgraaf L.C., de Bont L.G., Boering G., Liem R.S. (1995). The structure, biochemistry, and metabolism of osteoarthritic cartilage: A review of the literature. J. Oral Maxillofac. Surg..

[32] de Bont L.G., Stegenga B. (1993). Pathology of temporomandibular joint internal derangement and osteoarthrosis. Int. J. Oral Maxillofac. Surg..

[33] Izzetti R., Carli E., Gennai S., Giuca M.R., Graziani F., Nisi M. (2024). Treatment outcomes in patients with muscular temporomandibular joint disorders: A prospective case-control study. Dent. J..

[34] Dworkin S.F. (1994). Perspectives on the interaction of biological, psychological and social factors in TMD. J. Am. Dent. Assoc..

[35] Dahlström L. (1993). Psychometrics in temporomandibular disorders: An overview. Acta Odontol. Scand..

[36] Ohrbach R., Spalj S., Katic V., Alajbeg I., Celebic A. Diagnostic Criteria for Temporomandibular Disorders: Assessment Instruments. Version 15 May 2016. [Dijagnostički Kriteriji za Temporomandibularne Poremećaje (DK/TMP) Instrumenti Procjene: Croatian Version 23 March 2021].

[37] Vrbanović E., Zlendić M., Trošelj K.G., Tomljanović M., Vuković Đerfi K., Alajbeg I.Z. (2023). Association of Oxidative-Stress-Related Gene Polymorphisms with Pain-Related Temporomandibular Disorders and Oral Behavioural Habits. Antioxidants.

[38] D’apuzzo F., Rotolo R.P., Fordellone M., Cuomo G., Jamilian A., Nucci L., Grassia V. (2023). Temporomandibular Disorders and Serological Tests in Patients with Rheumatoid Arthritis. Appl. Sci..

